# Effects of Empagliflozin‐Induced Glycosuria on Weight Gain, Food Intake and Metabolic Indicators in Mice Fed a High‐Fat Diet

**DOI:** 10.1002/edm2.475

**Published:** 2024-03-12

**Authors:** Anh T. Nguyen, Zachary Amigo, Kathleen McDuffie, Victoria C. MacQueen, Lane D. Bell, Lan K. Truong, Gloria Batchi, Sara M. McMillin

**Affiliations:** ^1^ Fred Wilson School of Pharmacy High Point University High Point North Carolina USA

**Keywords:** diabetes, FGF21, metabolism, SGLT2 inhibitor

## Abstract

**Background:**

Sodium glucose‐linked transporter 2 (SGLT2) inhibitors promote glucose, and therefore calorie, excretion in the urine. Patients taking SGLT2 inhibitors typically experience mild weight loss, but the amount of weight loss falls short of what is expected based on caloric loss. Understanding the mechanisms responsible for this weight loss discrepancy is imperative, as strategies to improve weight loss could markedly improve type 2 diabetes management and overall metabolic health.

**Methods:**

Two mouse models of diet‐induced obesity were administered the SGLT2 inhibitor empagliflozin in the food for 3 months. Urine glucose excretion, body weight, food intake and activity levels were monitored. In addition, serum hormone measurements were taken, and gene expression analyses were conducted.

**Results:**

In both mouse models, mice receiving empagliflozin gained the same amount of body weight as their diet‐matched controls despite marked glucose loss in the urine. No changes in food intake, serum ghrelin concentrations or activity levels were observed, but serum levels of fibroblast growth factor 21 (FGF21) decreased after treatment. A decrease in the levels of deiodinase 2 (Dio2) was also observed in the white adipose tissue, a primary target tissue of FGF21.

**Conclusion:**

These findings suggest that compensatory metabolic adaptations, other than increased food intake or decreased physical activity, occur in response to SGLT2 inhibitor‐induced glycosuria that combats weight loss, and that reductions in FGF21, along with subsequent reductions in peripheral Dio2, may play a role.

## Graphical abstract

Overweight mice that were chronically administered empagliflozin did not lose weight despite marked caloric loss in the urine. No changes in food intake nor activity levels were observed, but serum levels of the metabolic regulating hormone FGF21 were reduced in the empagliflozin group. These studies suggest that SGLT2 inhibitor therapy induces changes in the hormonal regulation of metabolism.

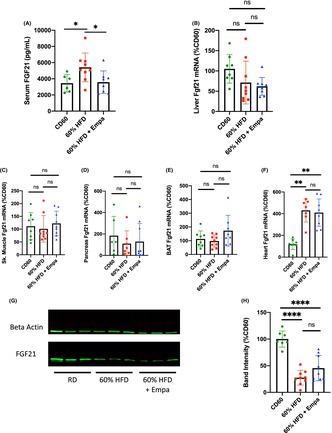

## 1 Introduction

Type 2 diabetes mellitus (T2DM) is a worldwide health epidemic affecting over 30 million people in the United States alone [1]. Complications from T2DM include retinopathy, neuropathy, nephropathy and increased risk for cardiovascular disease. Obesity is the single greatest risk factor for developing T2DM, and approximately 90% of patients diagnosed with T2DM are overweight or obese [1]. Accordingly, moderate weight loss (5%–10%) can improve glycaemic control and reduce the risk of T2DM‐related complications [2, 3, 4]. Therefore, optimising weight loss in patients with T2DM is of the upmost importance.

Sodium glucose‐linked transporter 2 (SGLT2) inhibitors, the newest class of medications approved for the treatment of T2DM in the United States, exhibit glycaemic‐lowering properties and beneficial cardiovascular and renoprotective effects. These medications work by blocking glucose and sodium reuptake in the proximal tubule of the kidney, resulting in glucose excretion in the urine (average of 60–90 g/day in T2DM patients [5, 6]). Because this glucose loss equates to a caloric loss (approximately 250–450 kcal/day), SGLT2 inhibitors are also associated with mild‐to‐moderate weight loss [5]. However, the observed weight loss with SGLT2 inhibitor use (~2–4 kg over 12 months) falls short of what is predicted based on the daily amount of calories excreted [7, 8, 9]. Mathematical models of human energy metabolism predict a compensatory increase in energy consumption to account for this discrepancy, and a small number of studies have documented such increases [10, 11, 12]. However, several other studies, including both rodent and human, have failed to support this explanation [13, 14, 15]. Most notably, the ENERGIZE study, which directly measured food intake in 54 participants, reported no differences in food intake between participants taking dapagliflozin and placebo [14]. Furthermore, despite conflicting data regarding compensatory changes in energy intake, few studies have investigated alternative explanations for the discrepancy between expected and observed weight loss with SGLT2 inhibitor use, such as potential decreases in energy expenditure.

In this study, we set out to investigate the effects of SGLT2 inhibitor‐induced glycosuria on body weight and the potential compensatory mechanisms to combat weight loss. We administered empagliflozin to two models of diet‐induced obese (DIO) mice and monitored weight gain, food intake, physical activity levels, as well as hormonal regulators of appetite (e.g. ghrelin) and metabolism (e.g. FGF21). We further investigated the effects of observed hormonal changes on metabolic gene expression in key target tissues.

## 2 Materials and Methods

### 2.1 Mouse Strains and Diet

All mice used in the study were male mice of the C57BL/6J strain (Jackson Labs, Bar Harbor, ME, USA). For the 60% high‐fat diet (HFD) studies, mice that had been fed a very high‐fat diet (60% HFD) since 6 weeks of age (C57BL/6J‐60%DIO; strain 380050) and their low‐fat diet controls (CD60; strain 380056) were obtained at 14 weeks of age and maintained on their respective diets. The 60% HFD (60% kcal from fat, 20% kcal from carbohydrates, 20% kcal from protein; Cat. No.: D12492) and the respective sucrose‐matched, low‐fat control diet (CD60; 10% kcal from fat, 70% kcal from carbohydrates, 20% kcal from protein; Cat. No.: D12450J) were purchased from Research Diets (New Brunswick, NJ, USA). For the 45% HFD studies, mice (strain 000664) were obtained at 3–4 weeks of age and placed on their respective diets immediately upon arrival. The 45% HFD (45% kcal from fat, 35% kcal from carbohydrates, 20% kcal from protein; Cat. No.: D12451) and the respective sucrose‐matched, low‐fat control diet (CD45; 10% kcal from fat, 70% kcal from carbohydrates, 20% kcal from protein; Cat. No.: D12450H) were also purchased from Research Diets. Mice were housed (3–5 per cage) in a controlled environment with a reversed 12:12‐h light:dark cycle. Experiments were conducted during the dark cycle under red light. Body weight measurements and blood draws were performed during the dark cycle in a biosafety cabinet using white light. The average temperatures in the animal room ranged from 21.3°C to 22.5°C, and the average humidity ranged from 36% to 41%. All animal experiments were conducted in accordance with policies of the NIH Guide for the Care and Use of Laboratory Animals and the Institutional Animal Care and Use Committee (IACUC) of High Point University.

### 2.2 Empagliflozin Administration

A subset of mice from each HFD group was subsequently placed on empagliflozin as described in Section 3. Mice were divided and housed in cages (2–3 cages per group) without a standardised randomisation procedure. Empagliflozin (Advanced ChemBlocks, Hayward, CA, USA) was incorporated into the HFD rodent chow at a concentration of 116.7 mg empagliflozin per 1 kg chow. This incorporation was accomplished by first pulverising the rodent chow with a mortar and pestle and spreading it over a large glass plate. The chow was then evenly sprayed with an empagliflozin solution that consisted of empagliflozin dissolved in 50% ethanol (11.67 mg/mL). The food was allowed to dry on the bench top for 30 min for ethanol evaporation and was then mixed with a 7% starch solution (tapioca starch in water; 2 mL solution per 100 g of chow) to reform the pellets. The control HFD chows used throughout this study were subjected to the same procedure, but empagliflozin was omitted from the 50% ethanol solution. The added starch (1.4 mg/g of chow) did not significantly change the caloric content (kcal/g) or the carbohydrate percentage of either HFD.

Mice were allowed to feed ad libitum. Each mouse consumed approximately 2.5–3 g of food per day delivering an approximate empagliflozin dose of 0.3–0.35 mg/day.

### 2.3 Food Intake Measurements

Weekly food intake was determined by weighing the amount of food added and measuring the amount of food remaining in each cage every week. Average daily food intake was calculated by dividing the grams of food consumed in a measurement period by the number of days in the period and the number of mice in the cage. Cumulative food intake was determined by adding the amount of food consumed for each cage each week and then dividing that sum by the number of mice in the cage.

### 2.4 Urine Collections and Urine Glucose Measurements

Tecniplast® metabolic cages (rodent cages; 23 cm interior diameter) were utilised to collect urine samples. Three mice (cage mates) were placed in each metabolic cage for a time period of 2 h/day on 3 consecutive days. Urine collections were performed at different times of the day for each of the 3 days (e.g. Monday 10:00–12:00; Tuesday 12:00–14:00; Wednesday 14:00–16:00). Urine was collected in the provided urine collection chambers, and the volume was recorded each day. Urine samples were transferred to microcentrifuge tubes and saved at −80°C for future analyses.

Glucose levels in the urine were determined using the Infinity® Glucose Reagent (ThermoFisher, Waltham, MA, USA) per manufacturer's instructions.

### 2.5 Body Weight Measurements

The body weight of each mouse was measured using portable laboratory scales on a weekly or bi‐weekly basis.

### 2.6 Locomotor Experiments

Total ambulatory counts were obtained using the Open Field Activity (Infrared Photobeam) chambers and the Activity Monitor software package (Med Associates®, Fairfax, VT, USA). Mice were placed in individual chambers (27.3 cm × 27.3 cm) and recorded for 30 min on two consecutive days. The ambulatory counts from Day 1 and the first 5 min of Day 2 (acclimatisation periods) were discarded. The ambulatory counts from the remaining 25 min from Day 2 were used for data analyses. Locomotor chambers were maintained in a dedicated quiet room with no disturbances during experiments.

### 2.7 Serum Collections From Live Mice

Serum samples were obtained from live mice by collecting blood from the tail vein. Mice were warmed via a heat lamp, and a small nick was made in the dorsal portion of the tail vein. Heparinised microhematocrit capillary tubes (Fisherbrand™) were used for blood collection. Blood samples were centrifuged at 3000*g* for 5 min in a tabletop centrifuge. Serum was removed via micropipette and was stored at −80°C for future analyses. For serum ghrelin measurements, mice were fasted overnight (12 h) before the first collection and were subsequently allowed to feed ad libitum (4 h) before the second collection.

### 2.8 Tissue and Serum Harvesting

Prior to tissue and serum harvesting, animals were fasted for 5 h, anaesthetised via isoflurane inhalation and sacrificed by cervical dislocation. Immediately following the death of the animal, blood was collected via cardiac puncture and immediately placed on ice. Tissues were removed and frozen on dry ice. Blood samples were subsequently centrifuged at 3000*g* for 5 min, and serum was collected and frozen at −80°C. Tissues were subsequently transferred to −80°C for long‐term storage.

### 2.9 Serum Hormone Measurements

Total ghrelin was measured in serum samples collected from live mice using the Rat/Mouse Ghrelin (total) ELISA Kit from Millipore Sigma® (Burlington, MA, USA) per manufacturer's instructions. For all other hormone measurements, serum collected during blood/tissue harvesting was used. Serum FGF21 and leptin levels were measured using the Mouse/Rat FGF‐21 Quantikine ELISA Kit and the Mouse Leptin DuoSet ELISA, respectively, from R&D Systems® (Minneapolis, MN, USA) per manufacturer's instructions. Serum glucagon and serum insulin were analysed using the Mouse Glucagon ELISA Kit and the Ultra‐Sensitive Mouse Insulin ELISA Kit, respectively, from Crystal Chem® (Elk Grove Village, IL, USA) according to the manufacturer's instructions.

### 2.10 RNA Isolations and Quantitative PCR Experiments

Messenger RNA was isolated from frozen tissues using the Purelink™ RNA Mini Kit (Thermo Fisher®) following the manufacturer's instructions. Synthesis of cDNA was performed using the iScript cDNA Synthesis Kit (BioRad®, Hercules, CA, USA), and quantitative PCR was performed utilising the iTaq SYBR green reagent (BioRad®), all per manufacturer's instructions. Beta 2 microglobulin was used as a reference gene. All primer sequences are listed in Table S1.

### 2.11 Protein Preparations and Western Blotting

Protein samples were prepared by homogenising frozen tissue samples in RIPA buffer (Thermo Fisher®) supplemented with SIGMA FAST® EDTA‐free protease inhibitor cocktail (Sigma®). Protein concentrations were determined via the Pierce BCA Protein Assay Kit (Thermo Fisher®), and 20 μg of each sample was run on a 4%‐ to 20%‐gradient polyacrylamide gel (BioRad®). Standard western blotting protocols were followed utilising nitrocellulose membrane (BioRad®). Primary antibodies were obtained from Invitrogen® (Anti‐FGF21; MA 5–35418) and Cell Signaling® (Anti‐Beta Actin; 4970). The anti‐rabbit IgG DyLight™ 800 (Cell Signaling®; 5151) was used as the secondary antibody, and blots were scanned with the LiCor Odyssey® infrared scanner. The Odyssey software was utilised for detection and band intensity calculations. Signal intensities of FGF21 were determined relative to those of beta actin.

### 2.12 Statistical Analyses

Graphing and statistical analyses were performed using the Graphpad Prism 8® software package. One‐ or two‐way analysis of variants (ANOVA) and Student's *t*‐tests with or without corrections for multiple comparisons were conducted as appropriate, and the specific analyses performed are indicated in each figure legend. Data were tested for normal distribution using the D'Agostino–Pearson (*n* ≥ 8) or Kolmogorov–Smirnov (*n* < 8) test. If data did not show normal distribution, further non‐parametric tests were performed to confirm statistical results and are indicated in the respective figure legends. Results were considered statistically significant when *p* < 0.05.

## 3 Results

### 3.1 Empagliflozin Causes Glycosuria Without Affecting Body Weight in Mice Fed a High‐Fat Diet

In order to induce metabolic disturbances similar to those observed in T2DM, we administered a diet that was very high in fat (60% kcal) to a group of C57BL6 male mice (60% HFD). Control mice were fed a protein‐matched control diet with a low‐fat content (10% kcal; CD60). After 12 weeks on these diets, mice receiving the HFD were subdivided into two groups and one was administered the SGLT2 inhibitor empagliflozin in the food at a concentration of 116.7 mg/kg food (60% HFD + Empa), which equated to an approximate dose of 0.3–0.35 mg/mouse day, a dose consistent with many published rodent studies [16, 17, 18, 19]. The other was maintained on the 60% HFD containing only vehicle and served as the HFD control group (60% HFD).

**FIGURE 1:**
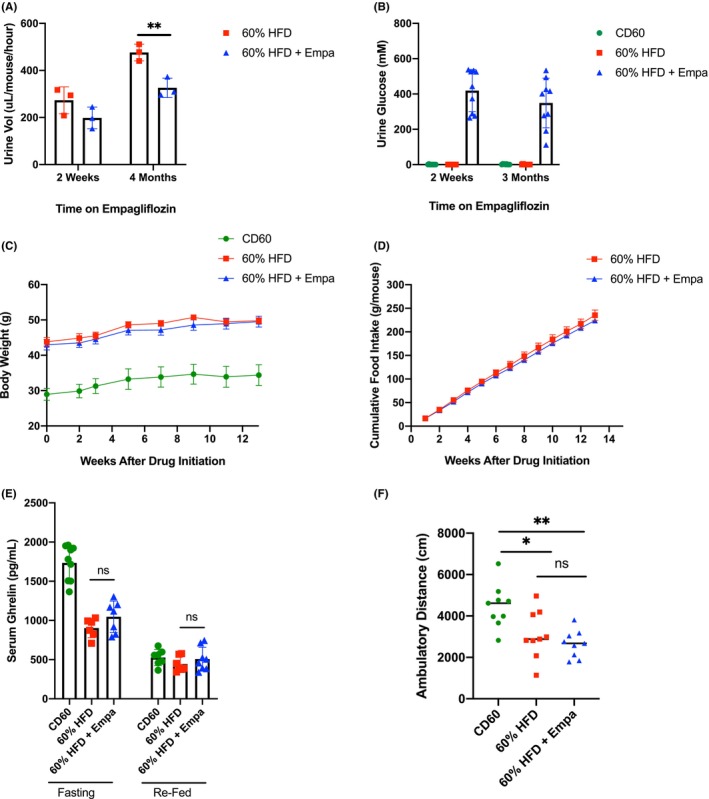
Effects of empagliflozin administration on mice fed a high fat diet. (A) Average urine output volume after 2 weeks and 4 months of empagliflozin administration; *n* = 3 pooled samples per group (three mice per pooled sample). Data were analysed by multiple *t*‐tests with the Holm–Sidak correction for multiple comparisons. (B) Average urine glucose concentrations after 2 weeks and 3 months of empagliflozin administration; *n* = 3 pooled samples per group (three mice per pooled sample). Collections and measurements performed in duplicate or triplicate. (C) Mouse body weights at baseline through 13 weeks of empagliflozin administration; *n* = 9 mice per group. (D) Cumulative food intake of the indicated mouse groups over the course of empagliflozin treatment; *n* = 3 cages per group (three mice per cage). Differences at Week 13 were analysed using Student's *t*‐test. (E) Serum ghrelin concentrations for the indicated mouse groups after 12 h of fasting and 4 h of ad libitum refeeding. Differences between the 60% HFD and 60% HFD + Empa groups were assessed by multiple *t*‐tests with the Holm–Sidak correction for multiple comparisons; *n* = 6–9 representative mice per group. (F) Total ambulatory distance travelled by the indicated mouse groups during 25‐min open‐field arena activity tests; *n* = 9 mice per group. Data were analysed using one‐way ANOVA with the Tukey test for multiple comparisons. Data in (A)–(E) are presented as mean ± SD. *p < 0.05; **p < 0.01.

Metabolic cages were utilised to collect urine samples. Urine volumes are represented in Figure 1A, showing volumes after 2 and 16 weeks on empagliflozin. Interestingly, overall urine volume increased over time for both the 60% HFD and 60% HFD + Empa groups (Figure 1A). While we did not observe a difference in urine volume for the 60% HFD + Empa group versus the HFD controls after 2 weeks, we observed an unexpected decrease in urine volume in the 60% HFD + Empa group compared with the 60% HFD controls (326 ± 41 vs. 476 ± 36 μL/mouse/h; *p* = 0.0087) after 16 weeks on empagliflozin (Figure 1A).

As expected, neither mice fed the CD60 control diet or 60% HFD alone had detectable levels of glucose in their urine. The 60% HFD + Empa group had an average urine glucose concentration of 419 ± 119 mM after 2 weeks on empagliflozin, and this did not significantly change over time (349 ± 141 mM after 4 months on empagliflozin; Figure 1B). Using these data, we calculated the average glucose loss in the urine to range from approximately 350 to 500 mg (1.4–1.9 kcal [5.7–8.1 kJ]) per mouse per day, which equates to approximately 10%–14% of daily caloric intake.

Body weights were measured at a minimum of every 2 weeks. Despite documented glucose/caloric loss via glycosuria, the HFD mice receiving empagliflozin did not exhibit significant weight loss compared with their HFD controls (Figure 1C).

### 3.2 Empagliflozin Does Not Affect Food Intake or Serum Total Ghrelin Levels

To determine whether caloric loss in the urine promoted a compensatory increase in appetite and food consumption in the 60% HFD + Empa group, we measured weekly food intake and assessed the levels of circulating ghrelin, the only known orexigenic hormone [20]. Mice in the 60% HFD control group consumed an average of 2.61 ± 0.15 g food (13.7 ± 0.8 kcal; 57.3 ± 3.3 kJ) per day while the mice in the 60% HFD + Empa group consumed 2.51 ± 0.18 g food (13.2 ± 0.9 kcal; 55.2 ± 3.8 kJ) per day. Similarly, the cumulative food intake of either mouse group did not significantly differ over the course of the study (Figure 1D). Serum total ghrelin measurements showed the expected increase in the fasting state versus the refed state for all mice, but the increase was blunted for mice maintained on a HFD (both groups; Figure 1E), which has been previously reported in the literature [21]. However, we did not observe a difference in total ghrelin levels between the 60% HFD + Empa group and the 60% HFD controls in either state (Figure 1E). These data suggest that the discrepancy between caloric loss and weight gain patterns among the 60% HFD and 60% HFD + Empa groups cannot be explained by increased appetite and/or caloric consumption.

### 3.3 Empagliflozin Administration Does Not Affect Mouse Locomotor Activity

To determine whether changes in physical energy expenditure could account for discrepancy, we investigated mouse activity levels by measuring ambulatory movement in mouse open field arena locomotor chambers. As expected, mice that had been fed a 60% HFD exhibited reduced activity levels compared with the CD60 controls (Figure 1F). However, the administration of empagliflozin did not significantly alter ambulatory movement when compared to the 60% HFD controls (Figure 1F).

### 3.4 Similar Results Observed in Mice Fed a High‐Fat/High‐Carbohydrate Diet

To determine whether similar findings would be observed in mice fed a diet high in carbohydrates, the studies were repeated with a separate cohort of mice administered a diet both high in fat (45% kcal from fat) and in carbohydrates (35% kcal from carbohydrates; 45% HFD). A low‐fat diet (CD45) control group was maintained on the protein‐matched, low‐fat diet (10% kcal from fat). After 12 weeks on the diet, the 45% HFD mouse group was subdivided, and half were administered the SGLT2 inhibitor empagliflozin in the food, at a concentration of 116.7 mg/kg food (45% HFD + Empa). The remainder continued on the 45% HFD containing only vehicle (45% HFD).

**FIGURE 2:**
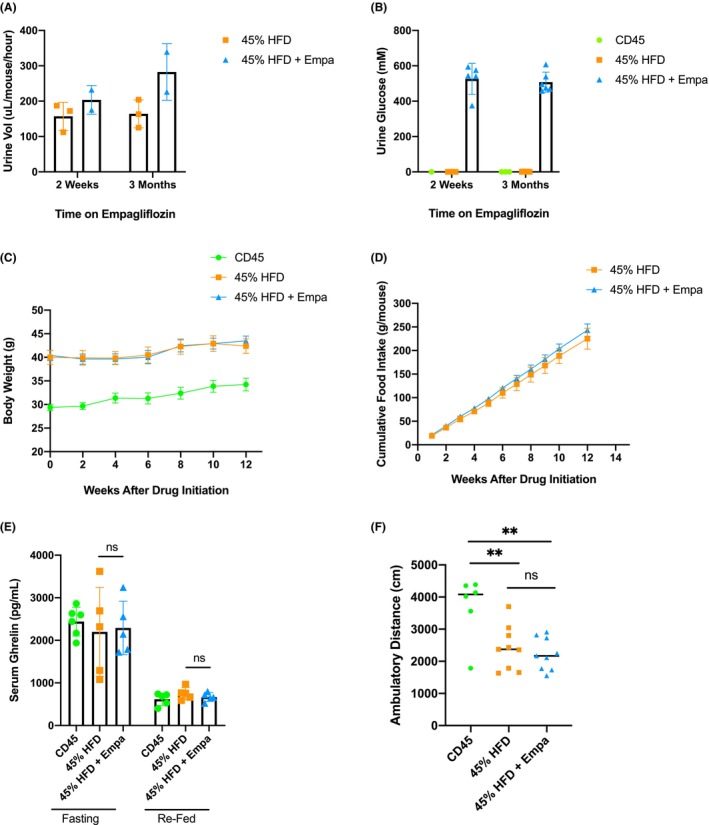
Effects of empagliflozin administration on mice fed a diet high in fat and carbohydrates. (A) Average urine output volume after 2 weeks and 3 months of empagliflozin administration; *n* = 3 pooled samples per group (three mice per pooled sample). Data were analysed by multiple *t*‐tests with the Holm–Sidak correction for multiple comparisons. (B) Average urine glucose concentrations after 2 weeks and 3 months of empagliflozin administration; *n* = 2–3 pooled samples per group (three mice per pooled sample); measurements performed in duplicate. (C) Mouse body weights at baseline through 13 weeks of empagliflozin administration; *n* = 15 mice per group. (D) Cumulative food intake of the indicated mouse groups over the course of empagliflozin treatment; *n* = 5 cages per group (three mice per cage). Differences at Week 13 were analysed using Student's *t*‐test. (E) Serum ghrelin concentrations for the indicated mouse groups after 12 h of fasting and 4 h of ad libitum refeeding. Differences between the 45% HFD and 45% HFD + Empa groups were assessed by multiple *t*‐tests with the Holm–Sidak correction for multiple comparisons; *n* = 5–6 representative mice per group. (F) Total ambulatory distance travelled by the indicated mouse groups during 25‐min open‐field arena activity tests; *n* = 6–9 mice per group. Data were analysed using one‐way ANOVA with the Tukey test for multiple comparisons. Data in (A)–(E) are presented as mean ± SD. *p < 0.05; **p < 0.01.

After 2 and 12 weeks on empagliflozin, there was no statistically significant difference in urine volumes between the 45% HFD + Empa group and the 45% HFD controls, and as expected, neither the CD45 control group nor the 45% HFD control group had measurable glucose in their urine at any time point (Figure 2A,B). Mice in the 45% HFD + Empa group had urine glucose levels of approximately 520 mM, and this did not significantly change over the course of the study (Figure 2B). Using these data, we calculated that the mice in the 45% HFD + Empa group lost approximately 450–600 mg of glucose per day in the urine which equates to approximately 1.7–2.3 kcal (7.3–9.7 kJ; ~13% to 17% of daily caloric intake).

As expected, mice receiving HFD maintained a higher body weight than the RD controls, but the 45% HFD + Empa subgroup revealed no significant differences in weight gain patterns compared with the 45% HFD controls (Figure 2C), similar to the results observed with the 60% HFD groups.

While we did observe a trend towards increased food intake in the 45% HFD + Empa group, this increase did not reach statistical significance (Figure 2D). As expected, serum total ghrelin levels were elevated in the fasting state versus the refed state for all mice (Figure 2E). Again, we did not detect any significant differences in serum ghrelin levels between the 45% HFD group and the 45% HFD + Empa group at either time point (Figure 2E).

Results from the locomotor chamber experiments showed no difference in ambulatory movement between the 45% HFD + Empa and 45% HFD control groups (Figure 2F).

Together, these results support the previous findings and suggest that metabolic compensations, other than increased appetite/food intake, or changes in physical activity were occurring in response to the caloric loss induced by empagliflozin.

### 3.5 Reduction in Serum Levels of FGF21 in Mice Receiving Empagliflozin

**FIGURE 3:**
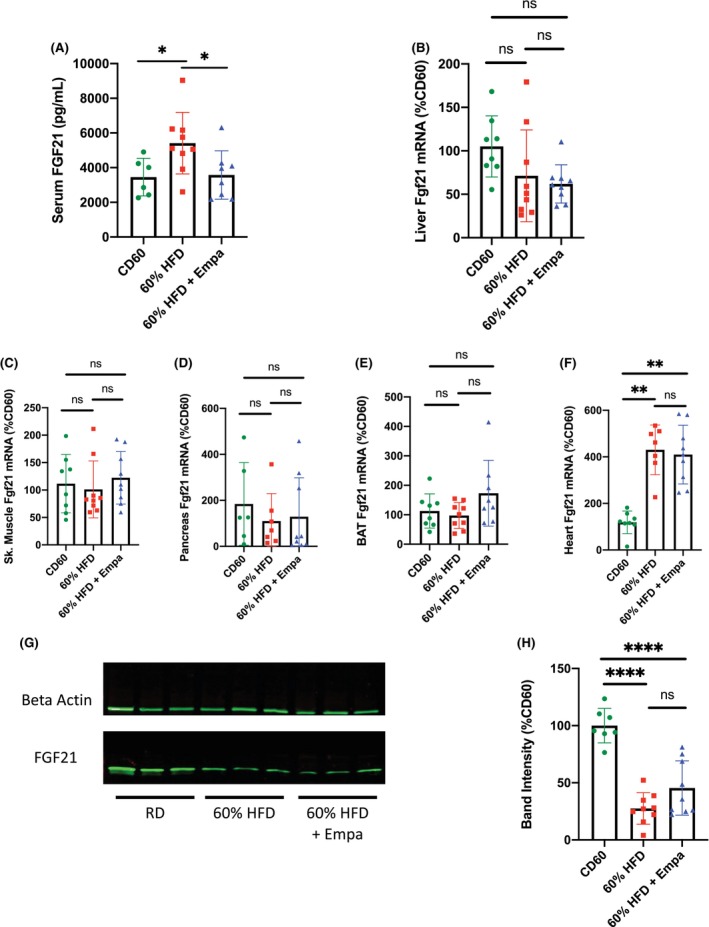
Analysis of FGF21 protein and mRNA levels in mice fed a high‐fat diet with and without empagliflozin. (A) FGF21 protein levels in serum samples collected from the indicated mouse groups; *n* = 6–9 mice per group. (B–F) FGF21 mRNA levels in the (B) liver, (C) skeletal muscle, (D) pancreas, (E) brown adipose tissue and (F) heart tissue isolated from the indicated mouse groups; *n* = 6–9 mice per group. (G) Representative western blot analysing FGF21 and beta actin protein levels in the liver tissue of the indicated mouse groups. Each of the three bands in each group is from three different mice. (H) Compiled band intensity data from three separate western blots analysing FGF21 levels in the liver tissue of the indicated mouse groups; FGF21 intensity was determined relative to beta actin intensity and comparisons are expressed as %CD60; *n* = 9 mice per group. Data were analysed by one‐way ANOVA with Tukey's correction for multiple comparisons. Due to non‐normal distribution of data in at least one group, (B)–(F) were also analysed via the non‐parametric Kruskal–Wallis test to confirm statistical results. Data are presented as mean ± SD. *p < 0.05; **p < 0.01; ****p < 0.0001.

Since locomotor experiments did not suggest that empagliflozin administration affected activity levels, we looked for signs of altered metabolic rate to potentially explain the discrepancy between calorie consumption/excretion and body weight balance. First, we measured serum levels of the hormone FGF21, an important master regulator of metabolism [22]. We observed significantly increased serum FGF21 with the administration of the 60% HFD (Figure 3A). Interestingly, the administration of empagliflozin blunted this response, resulting in significantly lower serum FGF21 levels in the 60% HFD + Empa group than in the 60% HFD controls (Figure 3A). The same trend was observed in the 45% HFD groups, although it did not reach statistical significance (Figure S1). Therefore, the remainder of the described investigations were carried out with the 60% HFD mouse groups.

### 3.6 FGF21 Expression Remained Unaffected by Empagliflozin Administration

To determine whether the reduced levels of FGF21 peptide in the serum were due to the changes in FGF21 gene expression, we measured the mRNA levels of FGF21 in liver tissue, the primary site of FGF21 synthesis and release [23]. Interestingly, we observed no statistically significant difference in Fgf21 mRNA in the livers of 60% HFD + Empa mice compared with their 60% HFD controls (Figure 3B).

Although not the primary site of synthesis and secretion for systemic circulation, FGF21 has been shown to be produced in various other tissues such as skeletal muscle, pancreas, brown adipose tissue (BAT) and heart [24]. To determine whether changes in serum FGF21 levels were potentially due to expression changes in one of these other tissues, we again performed quantitative PCR experiments to measure mRNA levels. No significant differences in Fgf21 mRNA levels were observed between any of the groups in skeletal muscle, pancreas or BAT tissue (Figure 3C–E). Interestingly, we observed a robust increase in Fgf21 mRNA in the heart tissue of mice fed a 60% HFD, but this increase was not affected by the administration of empagliflozin (Figure 3F).

Since the primary source of systemic FGF21 hormone is the liver, we analysed liver FGF21 peptide levels. Surprisingly, western blot experiments revealed a decrease in liver FGF21 peptide levels in mice fed a 60% HFD (Figure 3G,H). Although a number of mice in the 60% HFD + Empa group showed increased liver peptide levels relative to the 60% HFD controls, there was no significant difference in overall band intensity between these two groups (Figure 3H).

**FIGURE 4:**
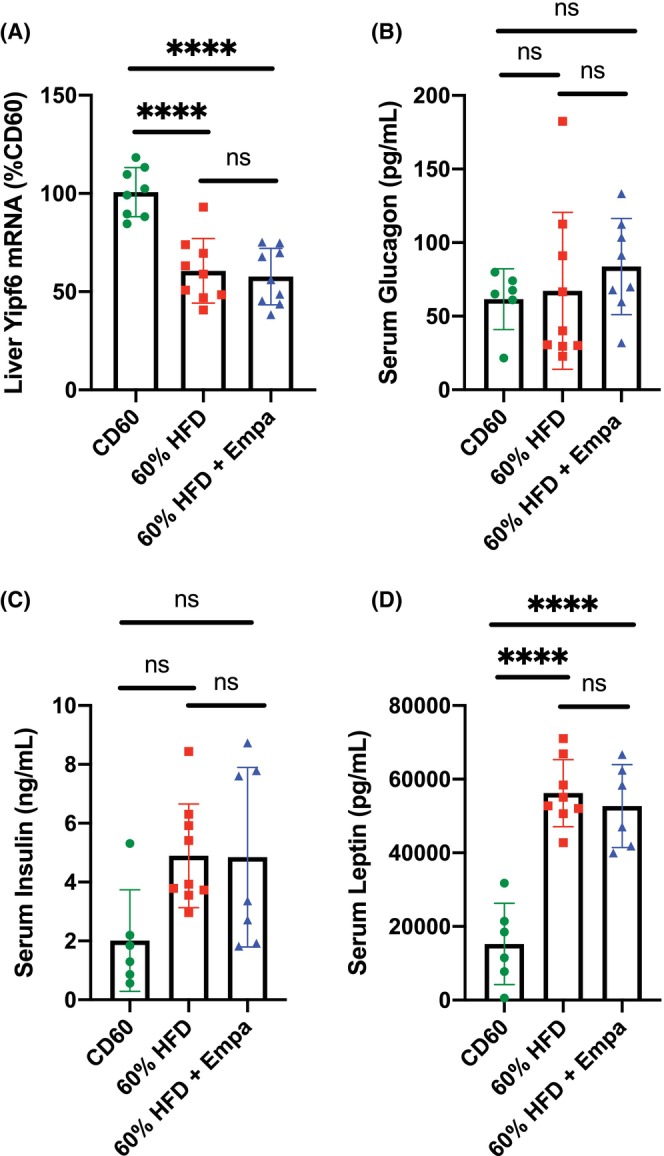
Investigation of liver YipF6 and serum glucagon levels in the HFD mice administered empagliflozin. (A) Relative Yipf6 mRNA levels in liver tissue of indicated mouse groups as determined by quantitative PCR; *n* = 8–9 mice per group. (B) Glucagon, (C) insulin and (D) leptin levels in serum samples collected from the indicated mouse groups; *n* = 6–9 mice per group. Data were analysed using one‐way ANOVA with Tukey's correction for multiple comparisons. Due to the non‐normal distribution of data in one group, (B) was also analysed using the non‐parametric Kruskal–Wallis test to confirm statistical results. All data are presented as mean ± SD. ****p < 0.0001.

Serum FGF21 levels are also affected by changes in FGF21 secretion from the liver, and FGF21 secretion has been shown to be negatively regulated by the Yip domain protein family member 6 (YipF6) [25]. We analysed the expression levels of YipF6 in the liver tissue of our mouse groups and found the mRNA levels of Yipf6 to be significantly decreased in the 60% HFD group compared with the CD60 controls (Figure 4A). However, there was no difference in liver Yipf6 mRNA levels between the 60% HFD and 60% HFD + Empa groups (Figure 4A). FGF21 levels have also been shown to be regulated by other metabolic hormones such as glucagon and leptin [26, 27]. We therefore measured the serum levels of glucagon, insulin and leptin, but found no significant differences in the levels of any of these hormones in the 60% HFD + Empa group compared with the 60% HFD controls (Figure 4B–D). Thus, the underlying cause of the difference in FGF21 serum levels between these two groups remains unexplained.

### 3.7 Beta Klotho, Fgf Receptor and Uncoupling Protein Expression was Unchanged While Dio2 Expression was Reduced in White Adipose Tissue of Mice Administered Empagliflozin

**FIGURE 5:**
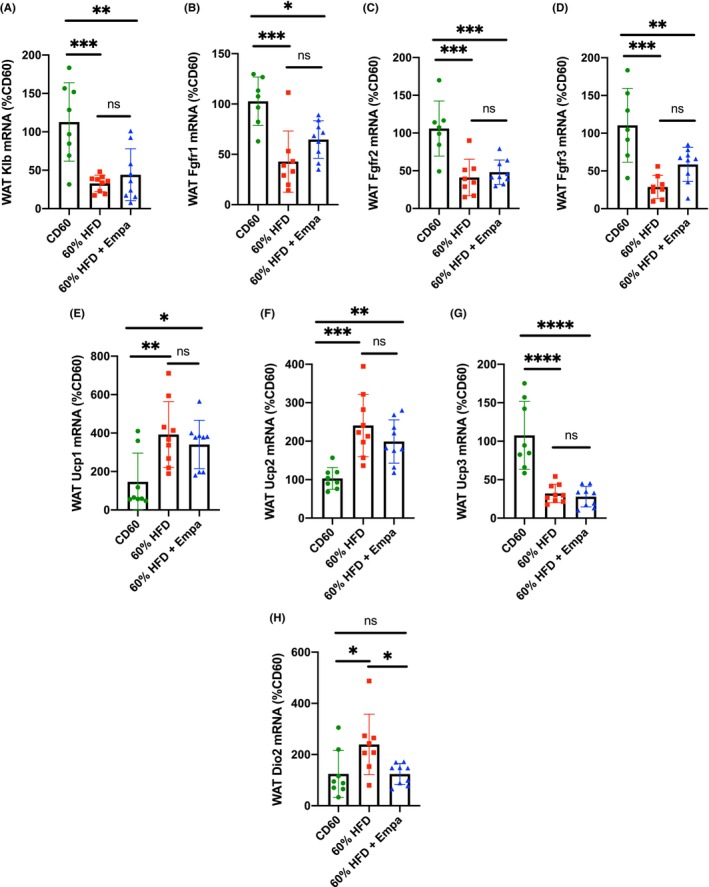
Analysis of gene expression in white adipose tissue. Relative mRNA levels of (A) beta klotho (Klb), (B) FGF receptor 1 (Fgfr1), (C) FGF receptor 2 (Fgfr2), (D) FGF receptor 3 (Fgfr3), (E) uncoupling protein 1 (Ucp1), (F) uncoupling protein 2 (Ucp2), (G) uncoupling protein 3 (Ucp3) and (H) deiodinase 2 (Dio2) in epididymal white adipose tissue (WAT) from the indicated mouse groups; *n* = 8–9 mice per group. Data are expressed as %CD60 controls and are presented as mean ± SD. Data were analysed using one‐way ANOVA with Tukey's correction for multiple comparisons. *p < 0.05; **p < 0.01; ***p < 0.001; ****p < 0.0001.

White adipose tissue (WAT) is a primary target for FGF21 signalling [23]. We therefore investigated the changes in WAT gene expression in our mice treated with empagliflozin. Beta klotho, the tissue‐specific component of the FGF21 receptor, is known to be reduced in WAT of mice fed a HFD, and this decreased expression could be an indicator of FGF21 resistance [23]. To determine whether the levels of beta klotho, and potentially FGF21 sensitivity, were altered by the administration of empagliflozin, we measured beta klotho mRNA in WAT. As expected, the mRNA levels were significantly reduced in our 60% HFD groups compared with the CD60 controls (Figure 5A). However, there was no significant difference between the 60% HFD and 60% HFD + Empa groups (Figure 5A). We also investigated the expression levels of several FGF receptors (Fgfr1–3) that interact with beta klotho to form the heterodimeric FGF21 receptor. All FGF receptor mRNA levels were decreased in the 60% HFD groups compared with CD60 (Figure 5B–D). The mRNA levels of FGF receptors 1 and 3 trended towards an increase in the 60% HFD + Empa group compared with the 60% HFD controls, but these differences did not reach statistical significance (Figure 5B,D).

We next looked at several known targets of FGF21 signalling that can affect metabolic rate [23]. While we did observe significant differences in the mRNA levels of uncoupling proteins 1–3 (Ucp 1–3) in mice fed a 60% HFD compared with CD60 controls, we did not observe any significant effect of empagliflozin administration (Figure 5E–G). Interestingly, the mRNA levels of Dio2, a key enzyme in the peripheral activation of thyroid hormone, were upregulated in the WAT of mice fed a 60% HFD but returned to the level of the CD60 controls in mice administered empagliflozin (Figure 5H), suggesting a link between metabolic rate and FGF21 signalling in mice maintained on empagliflozin.

## 4 Discussion and Conclusions

It is known that humans fail to lose as much weight during SGLT2 inhibitor use as is expected based on the documented caloric loss through glycosuria [10]. Mathematical models of human energy metabolism predict an increase in caloric intake to account for this discrepancy. However, human studies have failed to observe any increase in appetite and caloric intake [13, 14, 15]. Our findings, utilising two different DIO mouse models receiving empagliflozin, also fail to support this theory. Despite a marked caloric loss in the urine, we observed no weight loss or compensatory increases in food intake in mice receiving a HFD plus empagliflozin compared with their HFD controls. Furthermore, no reductions in physical activity levels were observed, suggesting that more complex alterations to metabolic rate are likely during SGLT2 inhibitor use. We note, however, that our methodology only monitored mouse movement or activity for short time periods (25 min) and acknowledge the possibility that more subtle differences in overall activity levels might be detected by continuous monitoring in the home cage.

It is well documented that decreases in resting energy expenditure occur upon diet‐induced weight loss in humans [28], and these shifts in metabolic rate are likely spurred by a negative energy balance [29, 30]. Although the exact molecular mechanisms are not completely understood, similar metabolic shifts may occur in response to the negative energy balance created by glycosuria during SGLT2 inhibitor use. We acknowledge the inability to estimate metabolic rate in this study, and that respiration data from indirect calorimetry would have been ideal.

In recent years, FGF21 has emerged as an important master regulatory hormone in metabolism. Its mechanisms of action are complex and incompletely understood. However, one study conducted with rodents has shown that prolonged FGF21 exposure increases energy expenditure and impacts weight control through actions on the central nervous system [22]. In agreement with published rodent and human studies [23, 31, 32], we found FGF21 hormone levels to be upregulated in the serum of mice fed a 60% HFD. Interestingly, we observed that mice maintained on empagliflozin were protected from this FGF21 increase, which may play a role in the weight loss discrepancy described above.

The receptor for FGF21 consists of an FGF receptor coupled to the single pass transmembrane protein beta klotho. Beta klotho has been shown to be reduced by a HFD, and this reduction has been proposed to underly a state of ‘FGF21 resistance’ [23]. In theory, such resistance could trigger compensatory increases in serum FGF21. As expected, we observed a decrease in beta klotho levels in the adipose tissue of our 60% HFD mice. However, we did not observe any difference in beta klotho mRNA levels between our 60% HFD and 60% HFD + Empa groups. FGF receptors 1, 2 and 3 also showed reduced expression in WAT of mice maintained on the 60% HFD, and while the mRNA levels of Fgfr1 and Fgfr3 trended towards an increase in mice administered empagliflozin, these differences did not reach statistical significance. Therefore, our data do not support a theory of empagliflozin relieving HFD‐induced FGF21 resistance and thereby normalising levels of the peptide.

The differences in FGF21 levels do not appear to be from changes in FGF21 gene expression. The liver is the primary source of systemic FGF21, and mRNA levels of FGF21 were similar in the liver of all mouse groups. However, western blot experiments revealed reduced levels of FGF21 protein in the liver tissue of mice fed a HFD. Increased serum levels together with reduced hepatic protein levels could potentially reflect increased secretion of FGF21 from the liver. In support of this theory, the 60% HFD mice had reduced mRNA levels of the protein YipF6, which has been shown to sequester FGF21 intracellularly in hepatocytes [25]. However, neither the western blot results nor the YipF6 findings suggest that empagliflozin has any effect on FGF21 secretion, leaving the difference in serum levels between 60% HFD and 60% HFD + Empa unexplained at present. It remains possible that mice on empagliflozin have higher rates of FGF21 degradation or clearance, and this should be the subject of future investigations.

A full examination of all FGF21 targets was beyond the scope of this study. However, since WAT is a primary target tissue for FGF21 signalling, we did investigate the expression levels of several metabolically relevant proteins that are known to be regulated by FGF21 [23] in the epididymal WAT depots of our mouse groups. While gene expression of UCP proteins remained unaffected by a 60% HFD or empagliflozin, we did see changes in the expression of Dio2, a deiodinase responsible for converting the thyroid hormone T4 into the highly active T3 form [23]. Dio2 expression has been shown to be upregulated by FGF21 [23]. In the present study, Dio2 mRNA levels were increased in WAT of mice fed a 60% HFD but were restored to baseline in those administered empagliflozin, a pattern that fits with relative FGF21 hormone levels. As Dio2 plays an important role in the availability of T3 within adipose tissue [33], a decrease in Dio2 would also correlate with reduced conversion of T4 to T3. As T3 is an important regulator of proliferation and metabolism, this finding creates an interesting link between empagliflozin, FGF21 and adipocyte metabolism.

The FGF21 findings in the current study also create an interesting link to current investigations in heart failure. SGLT2 inhibitor use is associated with robust benefits in heart failure, but these benefits remain largely unexplained [34]. Many studies have linked changes in FGF21 signalling to heart failure, and although some have suggested that FGF21 plays a protective role [35], other have shown increased FGF21 signalling to promote deleterious ventricular remodelling [36, 37]. While we did not observe an effect of empagliflozin on cardiac FGF21 expression in our mice fed a HFD, it remains possible that the reductions in the systemic circulation of FGF21 could play a role in heart health.

It should be noted that these studies were conducted in mice, and mouse metabolic shifts may not accurately reflect what occurs in humans. For example, it is well documented that patients receiving SGLT2 inhibitors do indeed lose weight (2–4 kg), albeit less than would be expected [10]. The fact that we observed no differences in weight gain in our mouse models is of potential concern. However, the resistance to weight loss in these mouse models might offer clues as to what is occurring in humans.

Despite the potential caveats, these studies suggest that metabolic alterations other than increases in appetite and food intake that occur during SGLT2 inhibitor induced glycosuria that counteracts weight loss. Reductions in the metabolic regulating hormone FGF21 and subsequent changes in gene expression, such as that of Dio2, may play a role in this shift and will be the focus of future studies. Given the current rate of obesity and T2DM, as well as the fact that weight loss improves glycaemic control and reduces associated co‐morbidities [2, 3, 4], there remains a compelling need to understand these metabolic changes in order to develop effective strategies to improve weight loss in patients with T2DM taking SGLT2 inhibitors.

## Author Contributions

**Anh T. Nguyen:** Conceptualization (supporting); formal analysis (supporting); investigation (equal); methodology (equal); writing – review and editing (equal). **Zachary Amigo:** Conceptualization (supporting); investigation (equal); methodology (supporting); writing – review and editing (supporting). **Kathleen McDuffie:** Investigation (equal); methodology (supporting); writing – review and editing (supporting). **Victoria C. MacQueen:** Investigation (equal); writing – review and editing (supporting). **Lane D. Bell:** Investigation (equal); writing – review and editing (supporting). **Lan K. Truong:** Investigation (equal); writing – review and editing (supporting). **Gloria Batchi:** Investigation (equal); writing – review and editing (supporting). **Sara M. McMillin:** Conceptualization (lead); formal analysis (lead); investigation (equal); methodology (lead); writing – original draft (lead); writing – review and editing (equal).

## Conflicts of Interest

The authors declare no conflicts of interest.

## Supporting information


Figure S1.

Table S1.

## Data Availability

The data that support the findings of this study are available from the corresponding author upon reasonable request.
