# Supplementary material for: Effects of Empagliflozin‐Induced Glycosuria on Weight Gain, Food Intake and Metabolic Indicators in Mice Fed a High‐Fat Diet
Source: Endocrinol Diabetes Metab. 2024 Mar 12;7(2):e00475. doi: 10.1002/edm2.475 (PMC10933387; doi:10.1002/edm2.475)
Supplement: Supplementary file 1 — Figure S1. [file EDM2-7-e00475-s002.zip › Supplement Figure caption.docx]

**Supplemental Figure 1. Serum FGF21 levels in mice fed a diet high in fat and carbohydrates.** FGF21 protein levels in serum samples collected from the indicated mouse groups as determined by ELISA; n = 6 - 9 mice per group. Data are presented as mean ± SD and were analyzed via one way ANOVA with Tukey’s correction for multiple comparisons.
