# Supplementary figures and images for: Effects of Empagliflozin‐Induced Glycosuria on Weight Gain, Food Intake and Metabolic Indicators in Mice Fed a High‐Fat Diet
Source: Endocrinol Diabetes Metab. 2024 Mar 12;7(2):e00475. doi: 10.1002/edm2.475 (PMC10933387; doi:10.1002/edm2.475)

Supplemental Figure 1

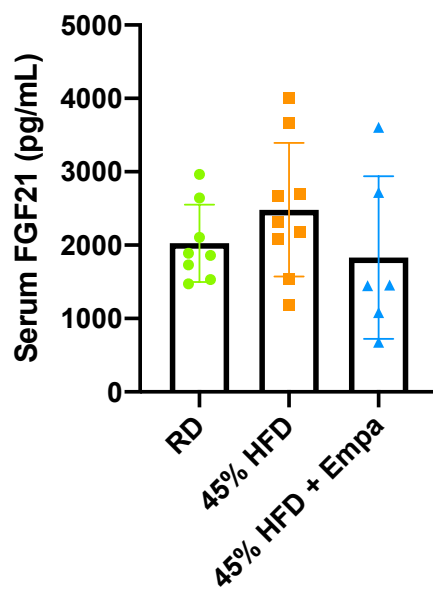

Supplement: Supplementary file 1 — Figure S1. [file EDM2-7-e00475-s002.zip › edm2475-sup-0001-FigureS1.pdf]
