# Supplementary material for: Effects of Empagliflozin‐Induced Glycosuria on Weight Gain, Food Intake and Metabolic Indicators in Mice Fed a High‐Fat Diet
Source: Endocrinol Diabetes Metab. 2024 Mar 12;7(2):e00475. doi: 10.1002/edm2.475 (PMC10933387; doi:10.1002/edm2.475)
Supplement: Supplementary file 2 — Table S1. [file EDM2-7-e00475-s001.docx]

**Supplementary Table 1**. Primer Sequences Used in Quantitative PCR Experiments.

| **Target Gene** | **Forward Primer** | **Reverse Primer** |
| --- | --- | --- |
| B2m | TCTCACTGACCGGCCTGTAT | ATTTCAATGTGAGGCGGGTG |
| Fgf21 | CCGCAGTCCAGAAAGTCTCC | CTGCAGGCCTCAGGATCAAA |
| YipF6 | CATCCAAGCCCCTGTTTGC | TCGCATAATGGTCCGACGAA |
| Klb | CTACACTGTGGGACACAACCT | AGCACAGAGGACATGGAGTG |
| Fgfr1 | ACTCTGGGGTTCTCCTGGTT | TCGCCAAGTGGTTTGCCTAA |
| Fgfr2 | TGTGGTCCCGTCAGACAAAG | TGGGCATCGCTGTAAACCTT |
| Fgfr3 | AGCGAGTTGTGCGGAGAG | CTGTACCATCCTTAGCCCAGAC |
| Ucp1 | CACGGGGACCTACAATGCTT | TAGGGGTCGTCCCTTTCCAA |
| Ucp2 | CGCCTTCTACAAGGGGTTCA | CGAGATTGGTAGGCAGCCAT |
| Ucp3 | GACCCACGGCCTTCTACAAA | TCAAAACGGAGATTCCCGCA |
| Dio2 | TGTCTGGAACAGCTTCCTCCT | AGTCAAGAAGGTGGCATTCGG |
